# Genomic alterations associated with loss of heterozygosity for *TP53* in Li-Fraumeni syndrome fibroblasts

**DOI:** 10.1054/bjoc.2000.1292

**Published:** 2000-07-24

**Authors:** E C Burt, L A James, M J Greaves, J M Birch, J M Boyle, J M Varley

**Affiliations:** CRC Cancer Genetics Group, Paterson Institute for Cancer Research, Wilmslow Road, Manchester, M20 4BX; CRC Paediatric and Familial Cancer Research Group, Royal Manchester Children’s Hospital, Manchester, M27 1HA

**Keywords:** Li-Fraumeni Syndrome, CGH, LOH, *TP53*, fibroblasts

## Abstract

Studies of Li-Fraumeni syndrome fibroblasts heterozygous for germline *TP53* mutations have shown that loss of heterozygosity (LOH) occurs during passaging and is associated with genomic instability, such as chromosomal aberrations and aneuploidy. to investigate the genomic changes associated with LOH in Li-Fraumeni (LF) fibroblasts, we have analysed cell strains at increasing population doublings (PD) using Comparative Genomic Hybridization (CGH). We have looked at three groups of cell strains: LF mutation-carrying strains which showed LOH for *TP53*, LF mutation-carrying strains which did not show LOH, and strains from normal individuals. Using CGH, we have detected loss of distinct chromosomal regions associated with LOH in 4 out of 5 mutation-carrying strains. In particular we have found loss of chromosomal regions containing genes involved in cell cycle control or senescence, including loss of 9p and 17p in these strains. Other recurrent changes included loss of chromosomes 4q and 6q, regions shown to contain one or more tumour suppressor genes. No genomic alterations were detected at cumulative PD in the normal strains or in the LF mutation-carrying strains which did not show LOH for *TP53*. We have also analysed the three groups of strains for microsatellite instability and somatic *TP53* mutations, and have found genetic alterations in only one strain. © 2000 Cancer Research Campaign


*British Journal of Cancer* (2000) **83**(4), 467–472
© 2000 Cancer Research Campaign
doi: 10.1054/ bjoc.2000.1292, available online at http://www.idealibrary.com on IDEAL®

### Genomic alterations associated with loss of heterozygosity for *TP53* in Li-Fraumeni syndrome fibroblasts

**EC Burt[1], LA James[1], MJ Greaves[1], JM Birch[2], JM Boyle[1] and JM Varley[1]**

[1]CRC Cancer Genetics Group, Paterson Institute for Cancer Research, Wilmslow Road, Manchester M20 4BX; [2]CRC Paediatric and Familial Cancer Research Group, Royal Manchester Children's Hospital, Manchester M27 1HA

**Summary** Studies of Li-Fraumeni syndrome fibroblasts heterozygous for germline *TP53* mutations have shown that loss of heterozygosity (LOH) occurs during passaging and is associated with genomic instability, such as chromosomal aberrations and aneuploidy. to investigate the genomic changes associated with LOH in Li-Fraumeni (LF) fibroblasts, we have analysed cell strains at increasing population doublings (PD) using Comparative Genomic Hybridization (CGH). We have looked at three groups of cell strains: LF mutation-carrying strains which showed LOH for *TP53*, LF mutation-carrying strains which did not show LOH, and strains from normal individuals. Using CGH, we have detected loss of distinct chromosomal regions associated with LOH in 4 out of 5 mutation-carrying strains. In particular we have found loss of chromosomal regions containing genes involved in cell cycle control or senescence, including loss of 9p and 17p in these strains. Other recurrent changes included loss of chromosomes 4q and 6q, regions shown to contain one or more tumour suppressor genes. No genomic alterations were detected at cumulative PD in the normal strains or in the LF mutation-carrying strains which did not show LOH for *TP53*. We have also analysed the three groups of strains for microsatellite instability and somatic *TP53* mutations, and have found genetic alterations in only one strain. © 2000 Cancer Research Campaign

**Keywords** Li-Fraumeni Syndrome; CGH; LOH; *TP53*; fibroblasts

The p53 protein was identified in 1979 through its interaction with viral oncoproteins (Lane and Crawford, 1979), and has subsequently been shown to play a central role in cell cycle control. The *TP53* gene is activated in response to DNA damage, resulting in the elimination of damaged cells by cell cycle arrest or apoptosis (reviewed in Wahl et al, 1997). The tumour suppressor gene *TP53* maps to chromosome 17p13.1, and is the most frequently altered gene in human cancer (Hollstein et al, 1991; Levine et al, 1991).

Li-Fraumeni syndrome (LFS) is an inherited cancer disorder, characterized by a broad but specific range of tumours, with germline mutations in *TP53* detected in approximately 70% of families (Li et al, 1988; Varley et al, 1997). Fibroblasts derived from LFS families have therefore been used to examine the mechanisms of tumorigenesis. Studies of fibroblasts heterozygous for germline *TP53* mutations have shown that loss of the wild-type allele, or loss of heterozygosity (LOH), occurs during passaging (or at increasing population doublings) and is associated with genomic instability, such as chromosomal aberrations and aneuploidy, and extension of proliferative lifespan (Rogan et al, 1995; Tainsky et al, 1995; Boyle et al, 1998).

Following on from our previous work looking at the genomic instability of LFS fibroblast strains at a chromosomal level, we have analysed changes at a molecular level. In particular we have studied chromosomal aberrations by Comparative Genomic Hybridization (CGH), and analysed microsatellite instability and somatic mutations in *TP53*.

CGH is a molecular cytogenetic technique which allows analysis of the entire genome for detection of chromosomal regions of DNA loss or gain. The technique is based on in situ hybridization and involves the differential labelling and co-hybridization of test (tumour or cell line) and reference (normal) DNAs to normal metaphase chromosomes. Developed in 1992 as a tool for cancer research (Kallioniemi et al, 1992), the technique has subsequently been refined to allow detection of regions of gain of 2 megabases, dependent on copy number increase, and regions of loss in the order of 10 megabases (Piper et al, 1995; Bentz et al, 1998).

We have used CGH to analyse mutation-carrying fibroblast cell strains derived from Li-Fraumeni families previously shown to undergo genomic instability during passaging (Boyle et al, 1998). In addition, we have analysed cell strains obtained from normal individuals, at increasing population doublings (PD).

### MATERIALS AND METHODS

Both LFS (Li et al, 1988) and LFL (Birch et al, 1994) cell strains have been classified using the general term Li-Fraumeni (LF, Boyle et al, 1998).

### Cell culture

All cell strains were established, expanded to senescence and *TP53* status characterized, as described previously (Boyle et al,

*Received 12 November 1999*
*Revised 1 March 2000*
*Accepted 23 March 2000*

*Correspondence to:* JM Varley

1998). Briefly, skin biopsies were obtained from LF family members and normal individuals, and used to set up fibroblast cell cultures. Cell cultures were expanded until senescence by repeated sub-culture, with cumulative PD calculated from cell counts. At intervals, aliquots from each cell strain were frozen down. Strain 163MA was separately cultured twice, producing the strains designated as 163MA-a and 163MA-b. High molecular weight DNA was extracted from cell aliquots using standard procedures.

### Comparative Genomic Hybridization

Cell strains were analysed by CGH, based on the method for high molecular weight DNA described recently (James, 1999), with minor modifications. Test (DNA from cell strains) and reference (normal male DNA; Sigma) DNAs were labelled with Rhodamine-dUTP (FluoroRed, Amersham) and FITC-dUTP (Spectrum Green, Vysis Ltd, Richmond, UK) respectively. Labelling was performed by nick translation to an optimum size of 200–3000 bp, with test and reference DNAs size-matched for each sample. Labelled DNAs (1 μg of each) and 50 μg Cot-1 DNA were combined, ethanol precipitated, and re-suspended in 10 μl of Hybridization Buffer (50% deionized formamide in 2× SSC/10% dextran sulphate). This probe mixture was denatured at 80°C for 8 minutes and pre-annealed for 30 minutes at 37°C, before applying to a target slide. Slides were prepared from PHA-stimulated, synchronized peripheral blood lymphocytes from a normal male donor, and denatured for 3–5 minutes at 72–75°C, depending on slide batch. Hybridization was carried out under a glass coverslip, in a moist chamber (50% formamide/2× SSC) at 37°C for 120 hours. Following hybridization, slides were washed to high stringency as described previously, and counterstained with an antifade solution containing DAPI.

### Image acquisition and analysis

Image acquisition and analysis were performed as described previously (James et al, 1997; James, 1999). Briefly, slides were viewed under a Zeiss Axioskop Fluorescence Microscope, and grey level images captured for each fluorochrome using a Photometrics CCD camera. Images were processed using Vysis Quips Software. At least 5 metaphases were analysed from each sample.

Each CGH experiment included a control slide with normal male DNA labelled as both test and reference. This slide was used to set fluorescence ratio threshold values, generally of 0.88 and 1.12, for analysis of cell strains. Any region exceeding these values was characterized as a loss or a gain respectively. Regions exceeding a value of 1.5 were defined as high level amplifications. Heterochromatic regions and the entire Y chromosome were excluded from analysis. CGH experiments were repeated on some samples to ensure reproducibility of results, and hybridization of normal male DNA vs. female DNA was also included as a control.

### Mismatch repair and microsatellite analysis

Two mononucleotide tracts (BAT26 and BAT40, Parsons et al, 1995) and four dinucleotide repeat sequences (D2S123, D3S1076, D8S255 and D13S175, Dib et al, 1996) were analysed. DNA was amplified in a 10 μl reaction mix containing reduced unlabelled dCTP and 1 μCi $^{32}$P-dCTP per reaction. All sequences were amplified for 35 cycles of 94°C, 56°C and 72°C each for 1 minute following an initial 4 minute denaturation at 94°C. Microsatellites were analysed by electrophoresis on a 6% denaturing acrylamide gel, and visualised by autoradiography.

### Mutation detection

Exons 5–8 of *TP53* were amplified from each sample and the products were screened initially by SSCP. Included in the analysis were the intron-exon boundaries. The primers which were used, together with the PCR conditions, are listed in Varley et al (1999). The products were separated on 0.5 × MDE gels (FMC Biochemicals) at 4–6 W for 12–16 h, dried and subjected to autoradiography. Controls (no DNA and normal DNA) were included on every gel. Any samples showing abnormal band shifts were re-analysed by repeating both first- and second-round SSCP–PCR reactions.

Wherever possible, early and late passage cells were analysed for both somatic mutation to *TP53* and microsatellite alterations.

### RESULTS

We have analysed three groups of cell strains by CGH: LF mutation-carrying strains which showed LOH for *TP53* (five strains), LF mutation-carrying strains which did not show LOH (three strains), and strains from normal individuals (five strains). All cell strains were analysed at regular intervals during expansion of cultures.

Aberrations detected by CGH, microsatellite changes and somatic mutations in *TP53* are summarized in Table 1, with *TP53* mutation and status indicated for each cell strain. Normal cell strains have not been tested for *TP53* mutations due to ethical constraints, hence *TP53* mutation and status are not shown.

### Comparative Genomic Hybridization

Four of five LF cell strains with LOH for *TP53* (135MA, 160MA, 161MA, 163MA) showed associated loss of several chromosomal regions, with loss in more than one cell strain affecting chromosomes 4q (minimal commonly lost region 4q33–35), 6q (6q15–21), 9 (9p; 9qcen–34.1), 16q and 17p (Figure 1B). The most common chromosomal losses associated with loss of heterozygosity for *TP53* were on the short arm of chromosomes 9 and 17, both present in at least three cell strains. Two independent sub-cultures of 163MA (a and b) both showed specific loss of 3q at late PD, in addition to the commonly affected regions of loss, with 163MA-b also showing additional changes, indicated in Table 1 and Figure 1A. Other cell strains in this group also showed specific regions of loss, such as 11p12–15 in 160MA and 8q in 161MA. Of the five strains analysed showing loss of heterozygosity for *TP53*, only one strain (21MA) did not show any associated chromosomal aberrations, detected by CGH. Only one region of gain was found in all the cell lines analysed, a high level amplification of 17q in strain 163MA-b at PD43.

LF mutation-carrying strains without LOH for *TP53* showed no chromosomal changes during passaging, when analysed by CGH. Similarly, none of the five normal strains showed any regions of loss or gain at any PD, when analysed by CGH. Loss of the *TP53* wild-type allele is associated with extension of cellular lifespan but does not generally result in more rapid growth.

© 2000 Cancer Research Campaign

**Table 1** Somatic mutation, microsatellite, and CGH analysis of normal and LF strains.

| Strain[a] | Family (mutation)[a] | PD[b] | *TP53* status[c] | *TP53* somatic mutations[d] | Microsatellite instability[e] | CGH analysis | |
|---|---|---|---|---|---|---|---|
| | | | | | | Losses | Gains[f] |
| Normal controls[g] | | | | | | | |
| 83MA | 7147 | 3.3 (9) | | | + | None | None |
| | | 26.8 (74) | | | + | None | None |
| 85MA | | 12.2 (29) | | | + | None | None |
| | | 27 (65) | | | | None | None |
| | | 41.8 (100) | | | + | None | None |
| 105MA | | 13.5 (47) | | | nd | None | None |
| | | 21.2 (74) | | | | None | None |
| | | 28.6 (100) | | | nd | None | None |
| 162MA | 5575 | 7.1 (27) | | | nd | None | None |
| | | 25.6 (98) | | | nd | None | None |
| 177MA | 2635 | 11.6 (36) | | | nd | None | None |
| | | 25 (79) | | | | None | None |
| | | 29.2 (92) | | | nd | None | None |
| Li-Fraumeni strains without LOH | | | | | | | |
| 110MA | 85 (E180K) | 13.8 (26) | +/mt | | | None | None |
| | | 22.8 (42) | +/mt | | | None | None |
| | | 31.8 (59) | +/mt | | | None | None |
| | | 40.8 (76) | +/mt | + | nd | None | None |
| | | 49.8 (92) | +/mt | nd | + | None | None |
| 191MA | 86 (SA I3) | 6.8 (16) | +/mt | nd | + | None | None |
| | | 17.4(40) | +/mt | | | None | None |
| | | 28.4 (65) | +/mt | + | + | None | None |
| 194MA | 64 (P152L) | 7.4 (16) | +/mt | + | + | None | None |
| | | 12.8 (28) | +/mt | | | None | None |
| | | 36.8 (80) | +/mt | | | None | None |
| | | 40.4 (89) | +/mt | + | + | None | None |
| Li-Fraumeni strains with LOH | | | | | | | |
| 21MA | 83 (R175H) | 4.1 (9) | +/mt | – (7) | + | None | None |
| | | 16.5 (37) | +/mt | | | None | None |
| | | 34.4 (78) | +/mt | -LOH(7) | LOH(1/6) | None | None |
| | | 36.0 (82) | +/mt | | | None | None |
| | | 37.0 (84) | –/mt | | | None | None |
| 135MA | 83 (R175H) | 6.3 (8) | +/mt | + | + | None | None |
| | | 19.5 (26) | +/mt | | | None | None |
| | | 33.7 (45) | +/mt | | | None | None |
| | | 48.9 (66) | +/mt | | | 4q33–35, 9, 16, 17p | None |
| | | 68.7 (93) | –/mt | + | + | 9, 16p, 17p | None |
| 160MA | 7003 (L344P) | 4.4 (26) | +/mt | nd | + | 6q13–21 | None |
| | | 13.0 (76) | –/mt | + | + | 1q21–41, 9p, 9q21–34.1, 11p12–15, 17p | None |
| 161MA | 7003 (L344P) | 8.0 (42) | +/mt | nd | + | 8q21.3–22 | None |
| | | 17.6 (93) | –/mt | + | + | 8q, 13, 17p | None |
| 163MA-a | 266 (R248W) | 7.8 (11) | +/mt | + | + | None | None |
| | | 25.2 (36) | +/mt | | | None | None |
| | | 45.7 (65) | +/mt | | | 9p | None |
| | | 65.8 (94) | –/mt | + | + | 3q, 9p, 17p | None |
| 163MA-b | 266 (R248W) | 15.4 (36) | +/mt | nd | nd | None | None |
| | | 29.2 (68) | +/mt | | | 4q34–qter | None |
| | | 43 (100) | –/mt | + | + | 3q, 4q32–qter, 6q15–22, 8p, 9p, 9qcen–34.1, 16q, 17p, Xq | **17q** |

[a]Strain and family numbers are as previously (Varley et al, 1997; Boyle et al, 1998; Boyle et al, 1999). [b]PD, population doubling. Figure in parenthesis shows the percentage of the maximum doubling time. [c]*TP53* status: +, wild type allele; – loss of the wild type allele (LOH); mt, mutant allele. [d]Somatic mutations of *TP53* exons 5–8, found by SSCP analysis: nd, not determined; +, normal; –, mutation detected with affected exon indicated in parentheses; LOH, loss of heterozygosity detected with affected exon indicated in parentheses. [e]Microsatellite analysis of 6 loci: nd, not determined; +, normal; –, microsatellite instability of one or more loci detected, with affected number indicated in parentheses; LOH, loss of heterozygosity of one or more loci detected, with affected number indicated in parentheses. [f]Amplifications are indicated by bold typeface. [g]Normal strains have not been tested for *TP53* mutations, therefore *TP53* status and somatic mutations are not shown for this group.

### Microsatellite analysis and somatic *TP53* mutations

All samples were analysed for microsatellite instability using a panel of six microsatellites. Only one sample showed an alteration (Table 1). At late passage 21MA showed loss of one allele of D3S1076. No sample showed any evidence of microsatellite instability.

All strains from patients with germline *TP53* mutations were analysed for additional somatic mutations and loss of the wild-type

© 2000 Cancer Research Campaign

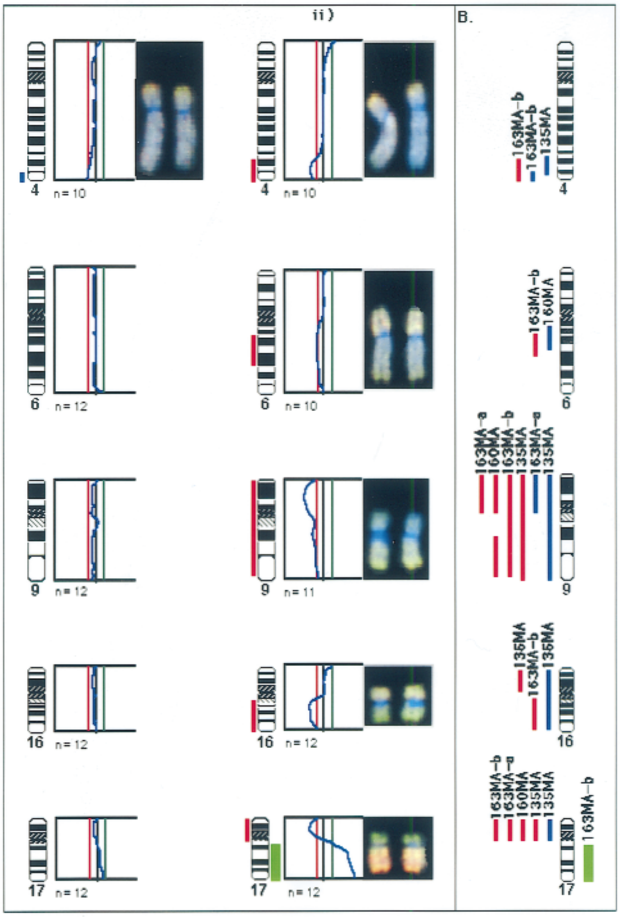

**Figure 1** Comparison of common DNA regions of imbalance detected by CGH in cell strains with LOH. The figure shows chromosomes 4, 6, 9, 16, and 17. (**A**) Cell strain 163MA-b at i) PD29 (before LOH) and ii) PD43 (after LOH), with fluorescence ratio profiles and corresponding CGH images of chromosomes shown. Hybridization is of Rhodamine-labelled test DNA and FITC-labelled reference DNA on to DAPI stained normal chromosomes. Chromosomal regions of loss appear green and regions of gain appear red on representative CGH images. On ratio profiles, red and green vertical lines correspond to threshold values of 0.88 (loss) and 1.12 (gain) respectively. The number of chromosome homologues (*n*) used to calculate the ratio is indicated below each profile. Chromosome ideograms are shown alongside the ratio profiles. Blue and red bars on the left side of each chromosome ideogram represent DNA losses before and after LOH respectively, whilst green bars on the right side represent gains after LOH (no gains were detected before LOH). Double-thickness green bars represent high level amplifications. (**B**) Summary of changes detected in two or more cell strains. The ideograms of each chromosome are shown, with coloured bars (blue, red or green) representing regions of DNA loss or gain as above. Specific cell strain numbers are indicated above each bar allele. This analysis was not carried out on strains from normal donors for ethical reasons. At early and mid passage strain 21MA showed an SSCP band-shift in exon 7, with loss of the corresponding normal sequence in the mid passage sample. This is interesting because there was no loss of the wild-type sequence corresponding to the germline mutation until late passage, indicating that there is a complex series of genetic alterations at the *TP53* locus in these cells. A similar situation has been reported in childhood tumours from patients with germline *TP53* mutations (Varley et al, 1999). Details of the samples with LOH are given in Table 1.

In cell strains with germline *TP53* mutations and no LOH, and in normal cell strains neither changes to the microsatellites tested nor somatic mutations to exons 5–8 of *TP53* were seen.

### DISCUSSION

Previously it has been shown that there is a strong correlation between loss of the wild-type *TP53* allele and chromosome instability, in cells from LFS families with germline *TP53* mutations (Boyle et al, 1998). It has also been postulated that this instability is the result of the conversion from a +/mt to a –/mt genotype. We have therefore analysed five cell strains from normal individuals and eight cell strains derived from LF families to assess genomic instability at a molecular level, and to dissect the events surrounding loss of the wild-type *TP53* allele. Amongst the LF cell strains, we have analysed both LF mutation-carrying strains with and without loss of heterozygosity for *TP53*, to deduce whether instability is the result of genotype conversion or a property of mutation-carrying strains irrespective of loss of the wild-type allele, as proposed by Tainsky et al (1995).

Using CGH, we have found loss of several distinct chromosomal regions associated with loss of the wild-type *TP53* allele in LF mutation-carrying strains, summarized in Table 1 and Figure 1B. In cell strains with LOH the most recurrent changes were loss of chromosomes 9p and 17p. It is likely that the targets for deletion in these regions could be genes involved in the control of cell proliferation and whose loss would confer a selective growth advantage on cells, such as tumour suppressor genes. Chromosome 17p contains the *TP53* gene and 9p harbours the *p16* tumour suppressor gene, on chromosome band 9p21. The *p16* gene (also known as *CDKN2*, *MTS1* and *INK4*), is inactivated in a variety of primary tumours and tumour cell lines (Kamb et al, 1994; Cairns et al, 1995). In addition an alternatively spliced transcript, encoding $p19^{ARF}$, is derived from the same locus as p16, and is therefore co-deleted in a number of malignancies (Mao et al, 1995; Quelle et al, 1995; Stone et al, 1995).

Interestingly, other work on LFS fibroblasts in culture has identified loss of both p16 and p53 function involved in the extension of proliferative lifespan and immortalization (Rogan et al, 1995; Noble et al, 1996). Whilst we have not detected immortalization in our set of LF strains, an increase in longevity (or proliferative lifespan) has been noted in these mutation-carrying strains compared to the control group (Boyle et al, 1998).

Other recurrent regions of loss have been detected by CGH, in particular loss of chromosomes 4q33–35, in cell strains 135MA and 163MA-b, and 6q15–21, in cell strains 160MA and 163MA-b (Figure 1). Microcell fusion studies have identified chromosomes containing senescence related genes, including chromosomes 4 and 6q (recently reviewed in Reddel, 1998). Inactivation of these regions has also been documented in several types of cancer, indicating the presence of one or more tumour suppressor genes. A potential tumour suppressor gene, *MORF 4*, has been identified in the region 4q33–34 (Bertram et al, 1999), although this has been excluded as the keratinocyte senescence gene (Bryce et al, 1999). A novel p53 target gene, *PA26*, has recently been characterized and localised to 6q21 (Velasco-Miguel et al, 1999), a region containing an unknown gene capable of inducing senescence (Morelli et al, 1997).

Whilst we have detected several regions of loss associated with LOH for *TP53* we have only found one associated region of gain, an amplification of the long arm of chromosome 17 in cell strain 163MA-b (shown in Figure 1). Gene amplification of 17q has been reported in a number of cancers, including breast and ovarian cancer (reviewed in Knuutila et al, 1998), and commonly involves the oncogene *ERBB2* at 17q21. Amplification of 17q22–24 is also

© 2000 Cancer Research Campaign a frequent event in breast cancer, and the gene *PS6K* has recently been localized to this region (Couch et al, 1999).

As well as detecting a number of recurrent chromosomal changes by CGH, we have also detected a number of strain specific changes, exemplified by the two independent sub-cultures of 163MA. Whilst both 163MA-a and 163MA-b showed loss of chromosomes 9p and 17p, seen in other cell strains with LOH, both sub-cultures also had loss of chromosome 3q at late PD, not seen in other strains. In addition, sub-culture 163MA-b also showed additional changes associated with LOH that 163MA-a did not exhibit (shown in Figure 1).

Overall, of the five mutation-carrying strains with LOH, only 21MA did not have any detectable chromosomal changes using CGH. This strain also did not show increased chromosomal aberrations associated with LOH, unlike other mutation-carrying strains (Boyle et al, 1998). However this was the only strain within the group that demonstrated changes at both a microsatellite locus and a somatic *TP53* mutation. Therefore 21MA appears to be atypical in having no chromosomal aberrations or CGH changes, but nonetheless does have genetic alterations.

None of the mutation-carrying strains without LOH for *TP53* had detectable chromosomal changes. These results support the model of genomic instability as a function of genotypic conversion rather than a property of mutation-carrying strains. However, using CGH we detected chromosomal changes prior to LOH for *TP53* in all the mutation-carrying strains with LOH. This result could be due to the sensitivity of LOH analysis, where the presence of a small proportion of +/mt cells against a background of –/mt cells can give a *TP53* status of +/mt. We also detected loss of chromosomal regions prior to LOH which could not be detected following LOH, confirmed by experimental repetition. One possible explanation could be that the first chromosomal change may become redundant on the appearance of subsequent changes, and may therefore be lost. Of the five control strains analysed by CGH at increasing PD, none showed any chromosomal changes, as expected.

In summary, we have shown that loss of distinct chromosomal regions is associated with LOH in LF cell strains, and involves regions containing genes involved in cell cycle control or senescence. It is likely that these changes are a function of genotypic conversion.

### REFERENCES

Bentz M, Plesch A, Stilgenbauer S, Dohner H and Lichter P (1998) Minimal sizes of deletions detected by comparative genomic hybridization. *Genes Chromosomes Cancer* **21**: 172–175

Bertram MJ, Berube NG, Hang-Swanson X, Ran Q, Leung JK, Bryce S, Spurgers K, Bick RJ, Baldini A, Ning Y, Clark LJ, Parkinson EK, Barrett JC, Smith JR and Pereira Smith OM (1999) Identification of a gene that reverses the immortal phenotype of a subset of cells and is a member of a novel family of transcription factor-like genes. *Mol Cell Biol* **19**: 1479–1485

Birch JM, Hartley AL, Tricker KJ, Prosser J, Condie A, Kelsey AM, Harris M, Morris Jones PH, Binchy A, Crowther D, Craft AW, Eden OB, Evans GR, Thompson E, Mann JR, Martin J, Mitchell ELD and Santibanez-Koref MF (1994) Prevalence and diversity of constitutional mutations in the p53 gene among 21 Li-Fraumeni families. *Cancer Res* **54**: 1298–1304

Boyle JM, Mitchell ELD, Greaves MJ, Roberts SA, Tricker K, Burt E, Varley JM, Birch JM and Scott D (1998) Chromosome instability is a predominant trait of fibroblasts from Li-Fraumeni families. *Br J Cancer* **77**: 2181–2192

Boyle JM, Greaves MJ, Camplejohn RS, Birch JM, Roberts SA and Varley JM (1999) Radiation-induced $G_1$ arrest is not defective in fibroblasts from Li-Fraumeni families without TP53 mutations. *Br J Cancer* **79**: 1657–1664

Bryce SD, Forsyth NR, Fitzsimmons SA, Clark LJ, Bertram MJ, Cuthbert AP, Newbold RF, Pereira-Smith OM and Parkinson EK (1999) Genetic and functional analyses exclude mortality factor 4 (MORF4) as a keratinocyte senescence gene. *Cancer Res* **59**: 2038–2040

Cairns P, Polascik TJ, Eby Y, Tokino K, Califano J, Merlo A, Mao L, Herath J, Jenkins R, Westra W, Rutter JL, Buckler A, Gabrielson E, Tockman M, Cho KR, Hedrick L, Bova GS, Isaacs W, Koch W, Schwab D and Sidransky D (1995) Frequency of homozygous deletion at p16/CDKN2 in primary human tumours. *Nat Genet* **11**: 210–212

Couch FJ, Wang X-Y, Wu G-J, Qian J, Jenkins RB and James CD (1999) Localization of PS6K to chromosomal region 17q23 and determination of its amplification in breast cancer. *Cancer Res* **59**: 1408–1411

Dib C, Faure S, Fizames C, Samson D, Drouot N, Vignal A, Millasseau P, Marc S, Hazan J, Seboun E, Lathrop M, Gyapay G, Morissette J and Weissenbach J (1996) A comprehensive genetic map of the human genome based on 5,264 microsatellites [see comments]. *Nature* **380**: 152–154

Hollstein M, Sidransky D, Vogelstein B and Harris CC (1991) p53 mutations in human cancers. *Science* **253**: 49–53

James LA (1999) Comparative genomic hybridisation as a tool in tumour cytogenetics. *J Pathol* **187**: 385–395

James LA, Mitchell ELD, Menasce L and Varley JM (1997) Comparative genomic hybridisation of ductal carcinoma in situ of the breast: identification of regions of DNA amplification and deletion in common with invasive breast carcinoma. *Oncogene* **14**: 1059–1065

Kallioniemi A, Kallioniemi O-P, Sudar D, Rutovitz D, Gray JW, Waldman F and Pinkel D (1992) Comparative genomic hybridization for molecular cytogenetic analysis of solid tumours. *Science* **258**: 818–821

Kamb A, Gruis NA, Weaver-Feldhaus J, Liu Q, Harshman K, Tavtigian SV, Stockert E, Day RS, Johnson BE and Skolnick MH (1994) A cell cycle regulator potentially involved in genesis of many tumour types. *Science* **264**: 436–440

Knuutila S, Bjorkvist A-M, Autio K, Tarkkanen M, Wolf M, Monni O, Szymanska J, Larramendy ML, Tapper J, Pere H, El-Rifai W, Hemmer S, Wasenius V-M, Vidgren V and Zhu Y (1998) DNA copy number amplifications in human neoplasms. *Am J Pathol* **152**: 1107–1123

Lane DP and Crawford LV (1979) T antigen is bound to a host protein in SV40-transformed cells. *Nature* **278**: 261–263

Levine AJ, Momand J and Finlay CA (1991) The p53 tumour suppressor gene. *Nature* **351**: 453–456

Li FP, Fraumeni JF, Jr Mulvihill JJ, Blattner WA, Dreyfus MG, Tucker MA and Miller RW (1988) A cancer family syndrome in twenty-four kindreds. *Cancer Res* **48**: 5358–5362

Mao L, Merlo A, Bedi G, Shapiro GI, Edwards CD, Rollins BJ and Sidransky D (1995) A novel $p16^{INK4A}$ transcript. *Cancer Res* **55**: 2995–2997

Morelli C, Sherratt T, Trabanelli C, Rimessi P, Gualandi F, Greaves MJ, Negrini M, Boyle JM and Barbanti-Brodano G (1997) Characterization of a 4-Mb region at chromosome 6q21 harboring a replicative senescence gene. *Cancer Res* **57**: 4153–4157

Noble JR, Rogan EM, Neumann AA, Maclean K, Bryan TM and Reddel RR (1996) Association of extended in vitro proliferative potential with loss of $p16^{INK4}$ expression. *Oncogene* **13**: 1259–1268

Parsons R, Myeroff LL, Liu B, Willson JK, Markowitz SD, Kinzler KW and Vogelstein B (1995) Microsatellite instability and mutations of the transforming growth factor beta type II receptor gene in colorectal cancer. *Cancer Res* **55**: 5548–5550

Piper J, Rutovitz D, Sudar D, Kallioniemi A, Kallioniemi OP, Waldman FM, Gray JW and Pinkel D (1995) Computer image analysis of comparative genomic hybridization. *Cytometry* **19**: 10–26

Quelle DE, Zindy F, Ashmun RA and Sherr CJ (1995) Alternative reading frames of the INK4a tumor suppressor gene encode two unrelated proteins capable of inducing cell cycle arrest. *Cell* **83**: 993–1000

Rogan EM, Bryan TM, Hukku B, Maclean K, Chang AC-M, Moy EL, Englezou A, Warneford SG, Dalla-Pozza L and Reddel RR (1995) Alterations in p53 and $p16^{INK4}$ expression and telomere length during spontaneous immortalization of Li-Fraumeni syndrome fibroblasts. *Mol Cell Biol* **15**: 4745–4753

Stone S, Jiang P, Dayananth P, Tavtigian SV, Katcher H, Parry D, Peters G and Kamb A (1995) Complex structure and regulation of the P16 (MTS1) locus. *Cancer Res* **55**: 2988–2994

Tainsky MA, Bischoff FZ and Strong LC (1995) Genomic instability due to germline p53 mutations drives preneoplastic progression toward cancer in human cells. *Cancer Metastasis Rev* **14**: 43–48

Varley JM, McGown G, Thorncroft M, Santibanez-Koref MF, Kelsey AM, Tricker KJ, Evans DGR and Birch JM (1997) Germ-line mutations of TP53 in Li-Fraumeni families: an extended study of 39 families. *Cancer Res* **57**: 3245–3252

*© 2000 Cancer Research Campaign* 

Varley JM, McGown G, Thorncroft M, James LA, Margison GP, Forster G, Evans DGR, Harris M, Kelsey AM and Birch JM (1999) Are there low penetrance TP53 alleles? Evidence from childhood adrenocortical tumors. *Am J Hum Genet* **65**: 995–1006

Velasco-Miguel S, Buckbinder L, Jean P, Gelbert L, Talbott R, Laidlaw J, Seizinger B and Kley N (1999) PA26, a novel target of the p53 tumor suppressor and member of the GADD family of DNA damage and growth arrest inducible genes. *Oncogene* **18**: 127–137

Wahl GM, Linke SP, Paulson TG and Huang L-C (1997) Maintaining genetic stability through TP53 mediated checkpoint control. *Cancer Surv* **29**: 183–219

*© 2000 Cancer Research Campaign*
